# Effect of herbal cream containing *Fumaria officinalis* and silymarin for treatment of eczema: A randomized double-blind controlled clinical trial

**DOI:** 10.22038/AJP.2022.19492

**Published:** 2022

**Authors:** Fariba Iraji, Behzad Sharif Makhmalzadeh, Mahya Abedini, Ali Aghaei, Amir Siahpoush

**Affiliations:** 1 *Skin Diseases and Leishmaniasis Research Center, Isfahan University of Medical Sciences, Isfahan, Iran*; 2 *Department of Pharmaceutics, Faculty of Pharmacy, Ahvaz * *Jundishapur University of Medical Sciences* *, Ahvaz, Iran*; 3 *Faculty of Pharmacy, Ahvaz Jundishapur University of Medical Sciences, Ahvaz, Iran*; 4 *Medicinal Plant Research Center, Ahvaz Jundishapur University of Medical Sciences, Ahvaz, Iran*

**Keywords:** Atopic dermatitis, Eczema, Herb, Silymarin, Fumaria officinalis, Silybum marianum

## Abstract

**Objective:**

Atopic dermatitis (AD) is a common skin disorder with symptoms including severe pruritus and eczematous lesions. AD affects between 5 and 20% of people in their life. Silymarin (SM) is a polyphenolic flavonoid from *Silybum marianum* L. and has several therapeutic characteristics including antiallergic, anticancer, and anti-inflammatory properties. *Fumaria officinalis* is a small plant that has a high antioxidant power and modulating effects on the immune system. Therefore, the current study intended to examine the influence of these two herbs extract on severity and symptoms of AD in patients.

**Materials and Methods:**

40 patients with mild to moderate eczema randomly received mometasone 0.1% or the herbal cream. Treatment course was 2 weeks and patients were examined before and after 2 weeks of treatment using the SCORAD system.

**Results:**

The reduction of SCORAD score was significant in both groups (p=0.04 in the herbal group and p=0.03 in the mometasone group) but no significant difference was observed between the groups. Mean SCORAD score was 27.66±5.9 before therapy and 4.77±1.6 after therapy in the mometasone group and mean SCORAD score was 26.05±7.1 before therapy and 6.944±2.6 after therapy in the herbal group.

**Conclusion:**

The current study indicated the impact of these two herbs extract on severity and symptoms of AD in patients; these plants may be a new treatment in reducing eczema symptoms and its problems.

## Introduction

Atopic dermatitis (AD) is a common skin disorder affects between 5 and 20% of people at some time in their life (Kantor R et al., 2016 ▶). Its main symptoms include severe pruritus and eczematous lesions. The main criteria to diagnose AD have been developed by Hannifin and Rajka (1979) ▶. These criteria represent the diversified manifestations of this disorder (Kantor R et al., 2016 ▶). AD is a complicated disease with several genetic and environmental causes (Vafaei-Nodeh et al., 2020 ▶). 

Primarily, epidermal barrier plays an important role in preventing this disorder; Nevertheless, several pathways are mentioned, so that its dysfunction increases the risk of AD. Some antigens can stimulate immunological reactions for instance, through affecting T-cells, which causes inflammation of the skin. Nevertheless, the influence of T helper 17 cells is well proven. According to the findings, Interleukin-17 may have a vital role in AD (Bozek et al., 2020 ▶). Still no treatment is available for AD. The majority of currently available therapeutic options (e.g. moisturizers) act based on reducing symptoms. Furthermore, for patients that suffer from chronic eczema, long-term application of some therapeutic interventions (e.g. topical and systemic corticosteroids) may cause side effects (Vafaei-Nodeh et al., 2020 ▶).

*Silybum marianum* L. (Milk thistle) has been used for a long-time to treat various disorders (Ashtiani et al., 2020 ▶). Silymarin is the main compound of the *S. marianum* and it has several benefits (Pickova et al., 2020 ▶). Silymarin (SM) is a polyphenolic flavonoid derived from the seeds of this plant and it has several therapeutic characteristics including antiallergic, anticancer, and antihyperglycemic functions (Lu et al., 2020 ▶). About 60 to 70% of SM is comprised of flavonolignans; silybin is the major ingredient (50–70% of SM composition). The other important ingredients are isosilybin, silydianin, silychristin, and isosilychristin (Vargas-Mendoza et al., 2020 ▶). SM has anti-inflammatory properties via NF-kB inhibition. In other words, SM inhibits NF-kB stimulation in response to Tumor necrosis factor alfa (TNF-α). This work is done by inhibiting the destruction of IKBa. Two general observed functions of NF-kB include: 1) Producing reactive oxygen species (ROS) production, especially H_2_O_2_ in places that have reduced antioxidant activity (Montalvo-Jave et al., 2008 ▶) and 2) presenting many pro-inflammatory factors such as cytokines, chemokines and enzymes (Banafsche et al., 2001 ▶). Silymarin reduces expression of T helper 1 (Th1) and increases expression the Th2 (Min et al., 2007 ▶) and can balance inflammatory factors IL-1 and IL-6 and dose-dependently increase IL-10 and IL-4 (Rainone, 2008 ▶).

*Fumaria officinalis* (FP), family Papaveraceae (Fumariaceae), is a small plant that is widely available in Eastern-Mediterranean countries. Historically, this plant is used for treating many inflammatory and painful illness (Raafat and El-Zahaby, 2020 ▶). It has antibacterial and antioxidant properties, mainly because of isoquinolinic alkaloids compounds (Păltinean et al., 2017 ▶). These compounds can be used for patients with minor hepatobiliary dysfunction, gastrointestinal disorders, cancer, and skin problems. FP has polyphenolic compounds like caffeic acid, rosmarinic acid, and apigenin, as well as alkaloids like chelerythrine and fumaritine (Adham et al., 2020 ▶). It is estimated that fumaric acid content of FP is nearly 0.93% w/w. Besides, it contains fumaric acid esters (FAEs). It is well-documented that FAEs can be used to treat psoriasis. The curative characteristics of FAEs are due to down regulation of type І cytokines secretion by T-helper lymphocytes, which means reduced secretion of interferon gamma (IFN-Y). It is reported that FAEs contain immunomodulatory properties such as inhibiting growth of T lymphocyte (Jowkar et al., 2011 ▶).

The aim of this study was to assess silymarin and *F. officinalis* combination as a therapeutic option for AD. Due to the high antioxidant power and these modulating effects on the immune system, these plants may be a new treatment in reducing the AD symptoms and its problems. Therefore, the current study intended to examine the impact of these two herbs extract on severity and symptoms of AD in patients.

## Materials and Methods

**Study design and population**

This research is a double‐blind controlled, parallel-group clinical trial on subjects with mild to moderate Eczema. The research protocols were confirmed by the Iranian Registry of Clinical Trials (IRCT20181026041466N5). 

According to previous trials, the sample size was calculated using 46-med-classic software and according to the following formula (n = (S12 + S22 ) ( Z1-a/2 + Z1-B )2/(X̅1 - X̅2)), considering the 95% confidence level and 80% test power, 20 patients in the intervention group and 20 patients in the control group were considered. (Bauer et al., 2011 ▶; Thouvenin et al., 2018 ▶; Sheehan et al., 1995 ▶) 40 subjects with mild to moderate eczema were investigated. The objectives of the study were explained to all participants before obtaining their written consent. Randomization was performed in individualized, block with statistical software. For randomization, the permuted block randomization was used with quadruple blocks produced by using the online site (www.sealedenvelope.com). The randomization list was available to the researcher and given to the participants, according to the order of the herbal cream (intervention group) or the mometasone 0.1% cream (control group). The blinding of the study was done in a way that the labels of the medicine containers were labeled by the researcher, so patients and physician were not aware of the used content. The data was given to the data analyzer by code A and B. 

Inclusion criteria: 1) All patients showing manifestation of eczema in a bilateral distribution. 2) All ages, both sexes, and different degrees of severity (mild, mild to moderate, and moderate) of eczema as assessed by the SCORAD index were included. 3) Patients received no topical or systemic treatment for at least 3 months before starting the study. 4) Written consent was obtained from all patients who participated in the present study.

**Medicines preparation**

*Fumaria officinalis* was purchased from reputable sources. After confirmation by a pharmacognosist, plants were powdered. Extraction was done using maceration method with ethanol 70 for 48 hr at room temperature following filtration. The freeze-drying method was used to remove the solvent and give a bulk dry powder. Silymarin was purchased from Iranian Institute of Medicinal Plants, Karaj, Iran. 

To prepare the herbal cream, components of oil phase including liquid paraffin, stearic acid, and cetyl alcohol were melted at about 60℃ and mixed with aqueous phase including water, Propylen glycol herbal extract, methyl paraben, glycerin, Tween 80.

The 0.1% mometasone cream (Megacort®, Kishmedipharm co, Iran) from the Iranian pharmaceutical market was used for the control group.

**Clinical trial**

In this trial, 40 patients referred to Sedighe tahere clinic of Isfahan, Iran, from 2020-10-06 to 2021-01-19 were randomly divided into two groups. Their information was recorded and they received a packet containing mometasone or the herbal cream. The treatment course was 2 weeks and patients were examined before and after 2 weeks for effect of medicine on SCORAD score and adverse effects such as erythema, irritation, itching, light sensitivity, intolerance to the drug, and other possible complications. After 2 weeks of treatment and follow up, clinical improvement outcomes and possible complications were examined by the physician. Application instruction was explained to patients. Participants were asked to softly wash their faces with confirmed cleanser and warm water, then gently patting their faces dry and then, apply the creams two times daily for 2 weeks. 

**Tests and evaluations**

**Measurement indices**

The European Task Force on Atopic Dermatitis (ETFAD) has developed the SCORAD (Scoring AD) index to create a consensus on assessment methods for AD (Oranje et al., 2007 ▶). To measure the extent of AD, the rule of nines is applied on a front ⁄back drawing of the patient’s inflammatory lesions. The extent can be graded 0–100. The intensity part of the SCORAD index consists of six items: erythema, edema excoriations, lichenification, oozing ⁄crusts and dryness. Each item can be graded on a scale of 0–3. The subjective items include daily pruritus and sleeplessness. Both subjective items can be graded on a 10 cm visual analogue scale. The maximum subjective score is 20. All items should be filled out in the SCORAD evaluation form. The SCORAD index formula is: A ⁄5 + 7B⁄2 +C. In this formula A is defined as the extent (0–100), B is defined as the intensity (0–18) and C is defined as the subjective symptoms (0–20). The maximum SCORAD score is 103 (Oranje et al., 2007 ▶).

**Statistical analysis**

Descriptive statistics such as frequency distribution, mean and standard deviation were used, and the Shapiro-Wilk test was used to investigate data normality. The two-sample independent T-test and chi-square test were used to compare the homogeneity of demographic characteristics and the severity of main variables between intervention and control groups in the baseline. The mean changes in measured variables were applied using statistical One-way Repeated measures ANOVA in order to evaluate the intra- and inter-group effects. All analyses were done with alpha less than 0.05 and 95% confidence interval to determine the significance level.

## Results

**Figure 1:**
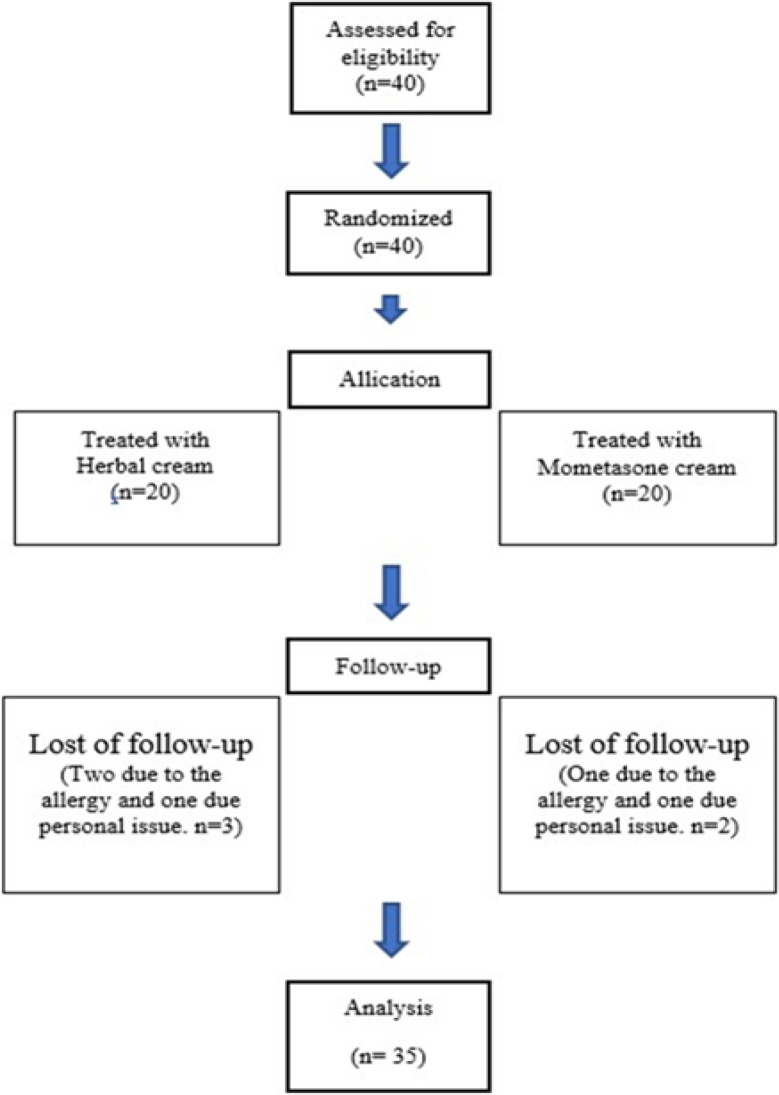
Flowchart of the study

In the present study, 40 patients with mild to moderate eczema were studied; among them, 31 were female and 9 were male. Two patients in the group receiving the herbal cream and 1 in the mometasone cream group were excluded due to allergy and two others (one in each group) were excluded due to the lack of proper use of drug and personal issues. Finally, 35 participants completed the study. No significant differences were observed between two groups for sex and age (p>0.05). Table 1 presents patients' demographic characteristics and Figure 1 shows the flowchart of the study.

**Table 1:** Patients’ demographic information

			Herbal group	Mometasone group
Gender	Male		5 (12.5%)	4 (10%)
			
		Female	15 (37.5%)	16 (40%)
Age		Under 20	2 (5%)	2 (5%)
		20-40	12 (30%)	10 (25%)
		Over 40	6 (15%)	8 (20%)

The reduction of SCORAD score was significant in both groups. It was (p=0.04) in the herbal group and (p=0.03) in the mometasone group. Mean SCORAD score was 27.66±5.9 before therapy and 4.77±1.6 after therapy in the mometasone group and mean SCORAD score was 26.05±7.1 before therapy and 6.944±2.6 after therapy in the herbal group. By use of independent T-test, no statistically significant improvement was observed in SCORAD score in the mometasone group compared with the herbal group. Table 2 presents the results of examination of the mometasone and herbal cream groups based on the type and number of lesions assessed by the physician.

**Table 2:** Comparison of scales during the treatment

**Type of medicine**	Mometasone				Herbal cream				**Comparison between two groups**
**Item** **(score)**	Before treatment	After 2 weeks	After 4 weeks	Change (%)	Before treatment	After 2 weeks	After 4 weeks	Change (%)	**Mometasone (%)** **-** **Herbal cream (%)**
**Erythema** **(0-3)**	1.391±0.43	0.347±0.07	0.181±0.016	40.31	1.8±0.37	0.6±0.21	0.444±0.12	45.18	**-4.86**
**Edema** **(0-3)**	0.782±0.21	0.130±0.04	0	26.08	0.6±0.14	0.4±0.13	0.333±0.10	8.88	**17.19**
**Oozing** **(0-3)**	0.347±0.08	0.130±0.05	0.454±0.17	10.07	0.1±0.02	0	0	3.33	**6.74**
**Dryness** **(0-3)**	2.217±0.47	0.782±0.21	0.5±0.25	57.24	2±0.58	0.7±0.19	0.555±0.17	48.14	**9.09**
**Lichenification** **(0-3)**	0.652±0.14	0.173±0.04	0.455±0.15	20.22	0.1±0.02	0.1±0.03	0.1±0.04	0	**20.59**
**Pruritus** **(0-10)**	5.304±2.1	0.861±0.34	0.130±0.37	46.22	4.5±1.38	1.7±0.47	1.222±0.35	32.77	**13.44**
**SCORAD** **(0-103)**	27.666±5.9	8.230±1.95	4.777±1.6	22.88	26.05±7.1	9.23±2.24	6.944±2.6	18.54	**3.67**

## Discussion

AD is a persistent, relapsing inflammatory skin disorder. Its main symptoms include pruritus, erythema, edema, and inflammatory eczematous eruptions (Lorz et al., 2020b ▶). Its prevalence is 15-30% in children and 10% in adults. However, these rates are increasing. Through reducing the self-esteem or disturbing the sleep pattern, AD may cause significant changes in the life of patients (Psomadakis et al., 2019 ▶). Several factors are reported to contribute to development of AD, e.g. family history, impaired skin barrier function, poor immune system, and environmental parameters (Lorz et al., 2020b ▶). Moreover, this disease may result in an imbalance between T helper cells 1 and 2. In AD, the predominant cell type is T helper 2; therefore, cytokines secreted by these cells (i.e. interleukin (IL)-4, IL-5, and IL-13) have an increased concentration in patients with AD, which has a significant impact on developing allergic responses. IL-5 causes eosinophil recruitment, while IL-4 and IL-13 are associated with antimicrobial peptides inhibition; enhanced concentration of IL-4 and IL-13 indicates the development of intensified skin inflammation. Moreover, IL-4 creates a positive feedback on T helper 2 cells (Lorz et al., 2020a ▶). To treat AD, based on the etiology and the severity of presentations, techniques like preventive strategies, topical anti-inflammatory treatment, and phototherapy can be applied. Use of steroidal ointment is the most common way of AD management. Nevertheless, prolonged use of steroids is associated with negative consequences like acne, steroid rosacea, and atrophy. However, in those who do not respond to steroid therapy, the probability of relapse is high (Lee et al., 2020 ▶). Although the prevalence of AD is high, but no effective treatment is developed for this disease (Vafaei-Nodeh et al., 2020 ▶). According to the literature, it can be concluded that silymarin and *F. officinalis* are associated with promising healing and restorative characteristics (Vrba et al., 2020 ▶). Antioxidant and anti-inflammatory properties of SM are well-established. It is reported that its antioxidant properties enhance the concentration of glutathione (Hamidian et al., 2020 ▶). Besides, it inhibits lipid peroxidation. In several countries, this substance is known as LegalonTM and HepatronTM (Hamidian et al., 2020 ▶). Zhao and colleagues found that silymarin has various influences on the skin (Zhao et al., 2000 ▶). Moreover, it was reported that SM prevents ultraviolet-B-stimulated skin injury (Gazak et al., 2007 ▶). Also, SM has a positive influence on healing wounds and skin burns caused by anti-inflammatory and antioxidant properties (Toklu et al., 2007 ▶). Furthermore, Han and colleagues, using an animal model, reported that SM could cause a suppressive impact on chemically-stimulated irritant contact dermatitis. Also, it was established that SM can prevent the expression of cytokines and infiltration of neutrophils; hence, it poses a significant impact on preventing and treating cutaneous inflammatory disorders (Han et al., 2007 ▶).

*F. officinalis* (FO) has an especial position in the Asian traditional medicine (Raafat et al., 2020 ▶). The efficacy and safety of FAEs are well proven. Dimethyl fumarate, the most important compound of FAEs, is a potent stimulator of T lymphocyte apoptosis (Jowkar et al., 2011 ▶). FAEs are potential therapeutic interventions for those who suffer from recalcitrant granulomatous skin diseases and it mediated by similar immunomodulatory pathways as in psoriasis (Litjens et al., 2006 ▶). Jowkar et al. (2014) ▶ investigated the effectiveness of topical fumaric acid 5% cream twice daily in comparison to triamcinolone 0.1% cream twice daily in 92 subjects. The results show that fumaric acid 5% can affect treating erythema. Although other symptoms improved with fumaric acid, but its effects were not better than triamcinolone 0.1%. Effect of triamcinolone 0.1% was better in decreasing excoriation, lichenification, edema, and eczema area and severity index (EASI) score (Jowkar et al., 2014 ▶).

This study indicated that combination of SM and FO extract can improve severity and symptoms of AD in patients and may be a new treatment in reducing eczema symptoms and its problems. Further studies are needed to introduce a product containing of SM and FO extract to pharmaceutical market as a good and new medicine for treatment of eczema with good effect and low adverse effect. 

## Conflicts of interest

The authors have declared that there is no conflict of interest.
